# Study on the effects of 4 × 4 min high−intensity interval training frequency on cardiorespiratory function, metabolic indices, and exercise performance in male university students

**DOI:** 10.3389/fphys.2026.1855612

**Published:** 2026-07-13

**Authors:** Hanwen Zhang, Yingjian Zhang, Heng Tian, Yang Gao, Mingxi Zhang

**Affiliations:** 1 Capital University of Physical Education and Sports, Beijing, China; 2 Langfang Normal University, Langfang, China; 3 Qufu Normal University, Qufu, China; 4 Army Engineering University of PLA, Xuzhou, China; 5 Hebei Sport University, Shijiazhuang, China

**Keywords:** aerobic endurance, high−intensity interval training (HIIT), maximal oxygen uptake (VO_2_max), running economy (RE), training frequency

## Abstract

**Objective:**

To determine the optimal weekly frequency of 4 × 4 min high−intensity interval training (HIIT) in male university students and thus provide evidence for prescribing efficient endurance training in this population.

**Methods:**

Forty−five physically active male university students were randomly assigned to three groups performing 4 × 4 min HIIT once, twice, or three sessions per week (n = 15 per group) for 6 weeks. Maximal oxygen uptake (VO_2_max), time to exhaustion, heart rate (HR), oxygen uptake (VO_2_), ventilatory threshold (VT), respiratory exchange ratio (RER), and blood lactate concentration were assessed via treadmill testing and breath−by−breath gas analysis. Repeated−measures ANOVA was used for statistical analysis.

**Results:**

After 6 weeks of intervention, all groups showed prolonged time to exhaustion and reduced submaximal HR and blood lactate, with the 2− and 3−session groups exhibiting larger improvement magnitudes. VO_2_max increased markedly in both the 2− and 3−session groups, while the 1−session group showed only a small increase. Submaximal VO_2_, VT, and RER changed minimally across groups. Post−intervention blood lactate was notably lower in the 2− and 3−session groups than in the 1−session group (p < 0.001), whereas physiological improvements were generally comparable between the 2− and 3−session groups. Training completion rates were 100%, 93%, and 80% in the 1−, 2−, and 3−session groups, respectively.

**Conclusion:**

Within the present study conditions, twice−weekly 4 × 4 min HIIT yielded substantial improvements in aerobic capacity and submaximal metabolic economy while maintaining relatively high adherence, showing a favorable balance between training benefits and tolerability. The twice−weekly frequency appears relatively optimal for physically active male university students. These findings are limited by the homogeneous sample and short intervention period, and further validation in larger, diverse cohorts is warranted.

## Introduction

High−intensity interval training (HIIT), characterized by alternating bouts of high−intensity exercise and recovery, has been widely demonstrated to markedly improve cardiorespiratory fitness and metabolic health within a relatively limited time frame (Gibala et al., 2012; Seiler et al., 2013; Mebler et al., 2018). Compared with traditional moderate−intensity continuous training, HIIT is considered more “time−efficient” for enhancing cardiorespiratory fitness, particularly maximal oxygen uptake (VO_2_max), promoting peripheral metabolic adaptations, and improving cardiovascular risk profiles, and has therefore attracted substantial attention in competitive sports, physical conditioning, and public health promotion (Helgerud et al., 2007; Weston et al., 2014; Venturini and Giallauria, 2022). Among the various HIIT prescriptions, the 4 × 4 min protocol represents a typical medium−to−long interval format. Owing to its clear structure, quantifiable workload, and good feasibility in both laboratory and applied settings, it has been widely adopted to elicit pronounced central and peripheral aerobic adaptations (Hawkins et al., 2007; Boullosa et al., 2020).

Maximal oxygen uptake (VO_2_max) is a central indicator of cardiorespiratory aerobic capacity and one of the key predictors of all−cause mortality and cardiovascular events (Blair et al., 1995; Kodama et al., 2009; Lee et al., 2010). In both athletic and clinical populations, 4 × 4 min HIIT has been shown to induce substantial increases in VO_2_max by repeatedly exposing the cardiovascular system to near−maximal oxygen demand and heart rate levels (Milanovic et al., 2015; Ramos et al., 2015). In addition to VO_2_max, time to exhaustion during incremental exercise testing provides an integrative measure of maximal exercise tolerance, reflecting not only central oxygen transport capacity but also peripheral muscular, metabolic, and perceptual factors (Batacan et al., 2017). Improvements in time to exhaustion following HIIT have been interpreted as indicating enhanced global endurance performance, encompassing both central and peripheral adaptations (Qi et al., 2026).

Endurance performance depends not only on the “ceiling” of aerobic capacity represented by VO_2_max, but also on the “efficiency of energy utilization” during submaximal exercise. VO_2_max reflects the capacity of the cardiopulmonary system to transport and utilize oxygen under maximal load. Running economy (RE), in contrast, emphasizes the energetic cost required to maintain a given running speed and is a critical determinant of middle− and long−distance performance as well as perceived tolerance during everyday exercise (Conley and Krahenbuhl, 1980). From a physiological perspective, under submaximal conditions, oxygen uptake (VO_2_) per unit speed, heart rate (HR) responses, ventilatory threshold (VT), respiratory exchange ratio (RER), and blood lactate concentration collectively characterize the cardiorespiratory and metabolic “cost” of sustaining a given external workload from different perspectives (Mandsager et al., 2018; Kaufmann et al., 2023; Oyarzo-Aravena et al., 2023; Benitez-Munoz et al., 2025). Fixed−speed VO_2_ reflects the oxygen cost of running at a given speed, HR reflects the circulatory effort required to support this workload, blood lactate provides insight into the balance between lactate production and clearance and the relative contribution of anaerobic glycolysis, VT marks the transition from predominantly aerobic to greater anaerobic contribution and underpins the intensity that can be sustained for prolonged periods, whereas RER reflects the relative contributions of fat and carbohydrate to energy provision at a given running speed. Consequently, VO_2_max and time to exhaustion alone are insufficient to fully capture the impact of HIIT on endurance capacity; a comprehensive evaluation should also incorporate RE and related circulatory and metabolic indices at submaximal intensities.

Accumulating evidence supports the efficacy of HIIT in improving VO_2_max and metabolic regulation across a wide range of populations, including individuals who are sedentary or metabolically compromised, generally active adults, and trained athletes (Batacan et al., 2017; Qi et al., 2026). By repeatedly driving HR and VO_2_ toward near−maximal levels, the 4 × 4 min HIIT protocol can concurrently induce adaptations in cardiac structure and function, improve vascular function, and augment skeletal muscle mitochondrial biogenesis and oxidative enzyme activity, thereby promoting multi−level aerobic remodeling (Milanovic et al., 2015). Nevertheless, in young males who already possess a certain training background, baseline VO_2_max and RE are often in the moderate−to−high range, leaving limited scope for further short−term increases in VO_2_max, and subtle metabolic “fine−tuning” at submaximal intensities may be difficult to manifest as large, rapid changes (Oleksandra et al., 2021). In this population, determining how to set an appropriate HIIT frequency that balances cumulative stimulus, recovery demands, and adherence—while still ensuring meaningful training effects—remains a relatively underexplored yet practically important issue.

Moreover, the patterns by which HIIT influences “central and peripheral adaptations” may not be identical across different training frequencies. Some studies have indicated that, within a certain range, increasing training frequency may confer additional “add−on effects” on peripheral metabolic adaptations such as mitochondrial volume density, expression of lactate transporters, and fat−oxidation capacity, which in turn may lead to lower blood lactate levels and better metabolic economy during submaximal exercise (Batterson et al., 2023). However, when training frequency is further elevated, marginal gains may diminish and, in some cases, accumulated fatigue and insufficient recovery may even attenuate certain adaptive processes (Campos et al., 2022). Within HIIT prescriptions, “training frequency” is therefore a key programming variable that may modulate the balance between physiological benefits and the demands on recovery and adherence. On the one hand, some studies suggest that performing HIIT once per week can already induce measurable improvements in cardiorespiratory function and metabolic risk factors, with adaptations exhibiting an approximately dose−dependent pattern across a frequency range of one to three sessions per week (Dalleck et al., 2010; Leung et al., 2024). On the other hand, there is evidence that excessive accumulation of training frequency and intensity may increase demands on recovery, elevate perceived fatigue, and potentially raise infection risk, thereby compromising adherence and long−term sustainability (Kavaliauskas et al., 2017; Souza et al., 2021). Therefore, when evaluating the effects of different HIIT frequencies, it is necessary to consider not only maximal capacity indices such as VO_2_max, time to exhaustion, and RE−related indices (fixed−speed VO_2_, HR, blood lactate, VT, and RER), but also whether indices reflecting circulatory and metabolic load at a fixed speed indicate a more economical response. At the same time, measures of training completion and adherence should be incorporated to obtain conclusions that are more relevant to real−world application.

In summary, research on the effects of different training frequencies of 4 × 4 min HIIT in young males with an established exercise background still presents several gaps. First, there is a lack of experimental data systematically comparing the impact of one, two, and three sessions per week, under a standardized protocol, on multiple dimensions including VO_2_max, time to exhaustion during maximal exercise, and submaximal indicators such as VO_2_, HR, VT, RER, and blood lactate. Second, few studies have simultaneously integrated “physiological benefits” and “training feasibility and adherence” within the same framework to explore optimization of HIIT frequency from a “benefit–burden balance” perspective. Against this background, the present study recruited male university students with regular exercise habits and implemented a 6−week intervention using an identical 4 × 4 min HIIT structure at three different weekly frequencies: once, twice, and three times per week. An incremental treadmill test and a steady−state RE test at 9 km/h were conducted to comprehensively assess changes in VO_2_max, time to exhaustion during maximal exercise, and fixed−speed VO_2_, HR, VT, RER, and blood lactate concentration, while training completion rates were recorded for each frequency condition. The aims were: (1) to compare the effects of different 4 × 4 min HIIT frequencies on cardiorespiratory aerobic capacity and circulatory and metabolic economy at submaximal intensities; and (2) to identify a relative balance point between physiological benefits and training adherence, thereby providing experimental evidence for the development of scientifically sound, efficient, and practically feasible HIIT−based endurance training prescriptions for physically active young adults.

## Methods

### Participants

*A priori* power analysis was performed using G*Power for repeated−measures ANOVA. Assuming an effect size of 0.3, an alpha level of 0.05, and a statistical power (1−β) of 0.80, a minimum sample size of 24 participants was required (Chinzara et al., 2022). Ultimately, 45 male university students from Capital University of Physical Education and Sports were recruited for this study. All participants had a continuous history of regular physical activity for at least 3 years, engaging in structured exercise at least three times per week (≥60 minutes per session), with consistent participation in their primary activities (resistance training, badminton, basketball, or soccer) for no less than 1 year prior to enrollment. Participants were classified as recreationally trained, defined as regular exercise participation without competitive athletic experience in the preceding 12 months.

Before participation, all subjects were informed of the study aims, experimental procedures, and safety precautions, and provided written informed consent. The study protocol was approved by the Ethics Committee of Capital University of Physical Education and Sports (Approval No. 2025A168). This trial was retrospectively registered at the Chinese Clinical Trial Registry (ChiCTR) on December 2, 2025, with registration number ChiCTR2500113733.

Participants were randomly assigned to one of three training-frequency groups using a computer-generated randomization sequence implemented in Matlab r2023(b). Allocation was concealed using sequentially numbered, sealed opaque envelopes opened only after baseline assessments were completed. Their baseline characteristics are presented in **Table 1**.

**Table 1:** Basic characteristics of participants.

Group	Age (years)	Height (cm)	Body mass (kg)
1/week	20.13 ± 0.99	176.00 ± 2.42	76.00 ± 2.90
2/week	20.33 ± 0.82	174.33 ± 4.10	75.33 ± 2.64
3/week	19.93 ± 1.44	175.53 ± 2.16	73.60 ± 4.00

### Experimental design

Participants were randomly assigned to one of three training−frequency groups according to the planned weekly HIIT frequency: 1 session/week, 2 sessions/week, or 3 sessions/week (n = 15 per group). There were no significant between−group differences in age, height, or body mass at baseline. The experimental protocol consisted of a pre−test, a 6−week training intervention, and a post−test. Both pre− and post−tests included a maximal oxygen uptake test and a RE test.

#### Maximal oxygen uptake test

Participants first completed at least 10 minutes of self−selected−speed warm−up running on a treadmill (Precor TRM833, USA), followed by 5 minutes of stretching. The formal test was then conducted while breath−by−breath gas exchange was recorded using a portable metabolic cart (META−LYZER 3B−R2, Cortex, Germany). Prior to each test, the system was calibrated with high−precision reference gases and a 3−L syringe. HR was continuously recorded using a Polar H10 heart rate monitor (Polar Electro Oy, Kempele, Finland).

The test protocol consisted of 5 minutes of steady−state running at 8 km/h, followed by incremental increases of 1 km/h every minute until volitional exhaustion. Treadmill incline was set at 1% throughout to simulate air resistance. Based on previous studies, VO_2_max was determined using indicators including the rating of perceived exertion (RPE), HR, and RER (Songli et al., 2023). All participants met the following criteria upon completion of the test: (1) rating of perceived exertion (RPE) of 19 or 20; (2) HR ≥ (220 − age) beats·min^-1^ and RER ≥ 1.10; and (3) a plateau in oxygen uptake, defined as no further increase in VO_2_ despite an increase in workload.

The speed at VO_2_max (vVO_2_max) was defined as the treadmill speed achieved at the point of VO_2_max during the incremental test. This vVO_2_max value from the pre-test was used to prescribe training intensity for the subsequent 6−week HIIT intervention.

#### Running economy test

Seventy−two hours after completion of the VO_2_max test, participants performed the RE test. After at least 10 minutes of warm−up running at 6–9 km/h and 5 minutes of stretching on the treadmill, the formal test commenced. Respiratory gas exchange was continuously measured using the same metabolic cart, and HR was recorded with a Polar HR monitor. Participants ran at a constant speed of 9 km/h for 8 minutes, with treadmill incline fixed at 1%. Immediately upon completion of the run, capillary blood was collected from the earlobe and analyzed for blood lactate concentration using a Lactate Pro 2 analyzer (Arkray KDK, Japan).

### Training intervention

The training intervention consisted of 4 × 4−minute high−intensity interval running. The 1−session/week group trained every Monday; the 2−sessions/week group trained on Monday and Wednesday; and the 3−sessions/week group trained on Monday, Wednesday, and Friday. In accordance with previous research, the intervention period was set at 6 weeks (Zinner et al., 2018).

During the first 2 weeks, participants were instructed to run each 4−minute interval at a speed corresponding to at least 85% of their individual vVO_2_max (speed at VO_2_max) determined during the pre−test incremental treadmill test, with target HR between 85% and 90% of maximal HR. Training intensity was progressively increased each week. By the final week, interval speed was required to reach at least 95% of VO_2_max running speed, with HR maintained between 90% and 95% of maximal HR. The recovery period between intervals was 4 minutes of active walking at 4 km/h. To ensure compliance with the prescribed intensity, all participants wore a Polar HR monitor during training sessions.

Outcome assessors were not blinded to group allocation. Given the specific nature of the exercise intervention, direct supervision of training sessions was required. This necessitated ongoing communication and familiarity between the research team and participants, and the training programs were fully disclosed to participants. Consequently, blinding was not feasible in this study.

### Outcome measures

In the pre- and post-tests of the VO_2_max test, outcome measures included VO_2_max and time to exhaustion. In the pre- and post-tests of the RE test, outcome measures included VO_2_, HR, VT, and RER during the final 10 seconds of the test, as well as blood lactate concentration measured immediately upon test completion.

### Statistical analysis

All statistical analyses were performed using SPSS 26.0 (IBM Corp., Armonk, NY, USA). Data are presented as mean ± standard deviation (M ± SD). The Shapiro–Wilk test was used to assess normality; all variables were normally distributed. A 2 (time: pre vs. post) × 3 (group: 1, 2, 3 sessions/week) repeated−measures ANOVA was conducted for each outcome variable. When appropriate, post−hoc comparisons were performed using the Bonferroni adjustment. Effect sizes were quantified using partial eta squared (η²). Statistical significance was set at p < 0.05.

## Results

Over the 6−week intervention period, four participants (one from the 2−session group and three from the 3−session group) withdrew due to an inability to tolerate the training load. Baseline characteristics of dropouts (mean ± SD): age 20.00 ± 1.63 years, height 173.00 ± 4.40 cm, body mass 72.25 ± 2.99 kg. Data from the remaining 41 participants were included in the final analyses. Group−specific completion rates were as follows: all 15 participants in the 1−session group completed the intervention (completion rate 100%); 14 participants completed the intervention in the 2−session group (completion rate 93%); and 12 participants completed the intervention in the 3−session group (completion rate 80%). Analysis was performed on completers only (N = 41).

### Maximal oxygen uptake test

#### Maximal oxygen uptake

**Figure 1:**
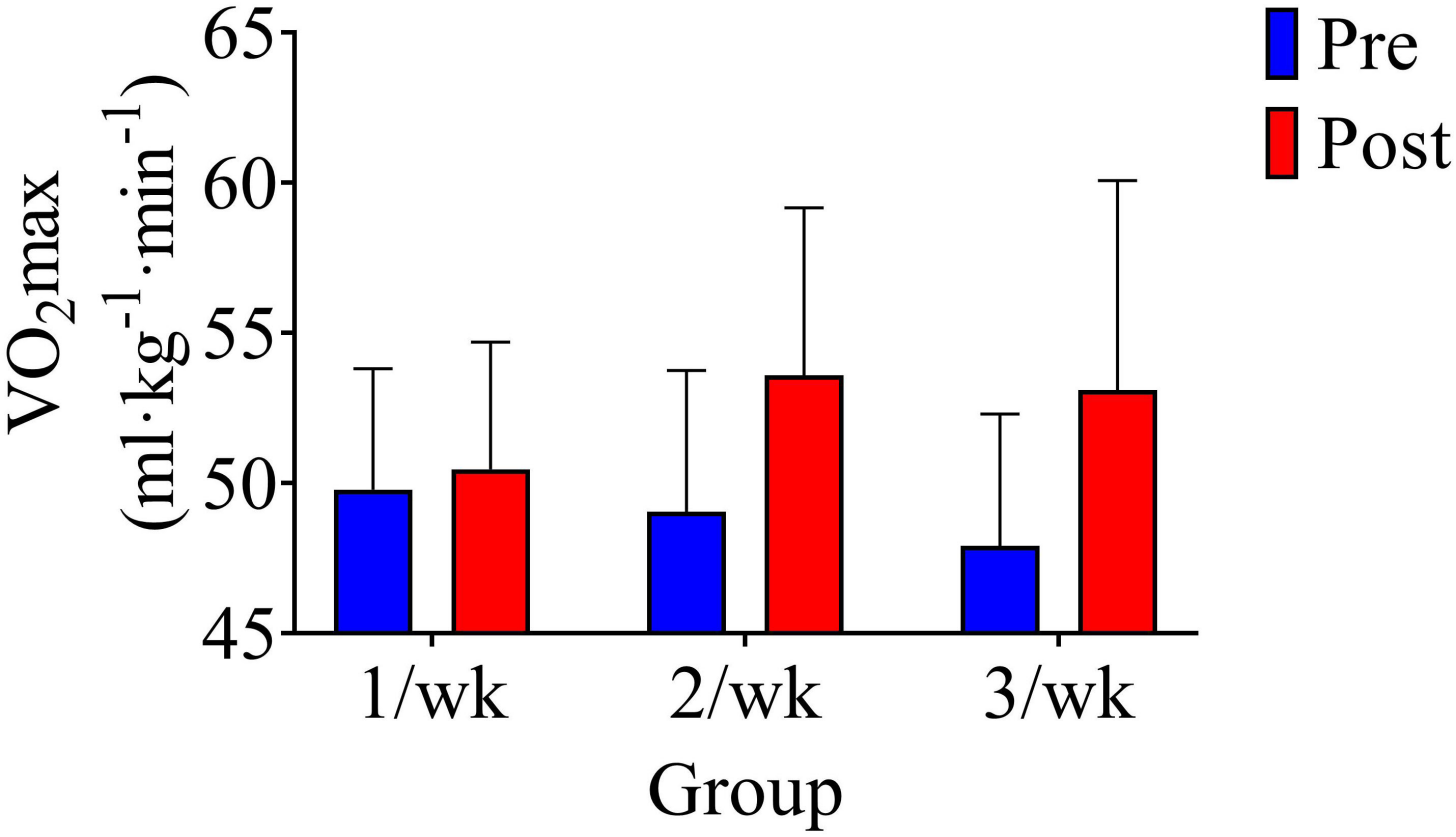
Changes in VO_2_max before and after the intervention in each group. 1/wk, 2/wk, and 3/wk indicate 1, 2, and 3 sessions per week, respectively; Pre indicates pre−test and Post indicates post−test.

VO_2_max outcomes are presented in **Figure 1**. The means ± SD before and after the intervention were as follows: in the 1−session group, 49.78 ± 4.03 ml·kg^-1^·min^-1^ pre−test and 50.46 ± 4.24 ml·kg^-1^·min^-1^ post−test; in the 2−session group, 49.04 ± 4.71 ml·kg^-1^·min^-1^ and 53.58 ± 5.58 ml·kg^-1^·min^-1^; and in the 3−session group, 47.90 ± 4.40 ml·kg^-1^·min^-1^ and 53.09 ± 6.97 ml·kg^-1^·min^-1^, respectively. Repeated−measures ANOVA revealed a significant main effect of time (F(1, 38) = 8.924, p = 0.005, η² = 0.190), with no significant main effect of group (F(2, 38) = 0.462, p =0.633, η² = 0.024) and no significant group × time interaction (F(2, 38) = 1.527, p = 0.230, η² = 0.074). *Post hoc* multiple comparisons with Bonferroni adjustment indicated that VO_2_max was significantly higher at post-test than at pre-test (p = 0.005).

Although baseline VO_2_max was numerically lower in the 3−session group compared with the 1− and 2−session groups, the difference did not reach statistical significance. This slight baseline discrepancy may reflect subtle differences in pre-training trainability, which could partially explain the relatively larger relative improvement observed in the 3−session group. Importantly, the absolute gains in VO_2_max were comparable between the 2− and 3−session groups, indicating that training frequency, rather than baseline fitness level, was the main determinant of aerobic adaptation.

#### Time to exhaustion

**Figure 2:**
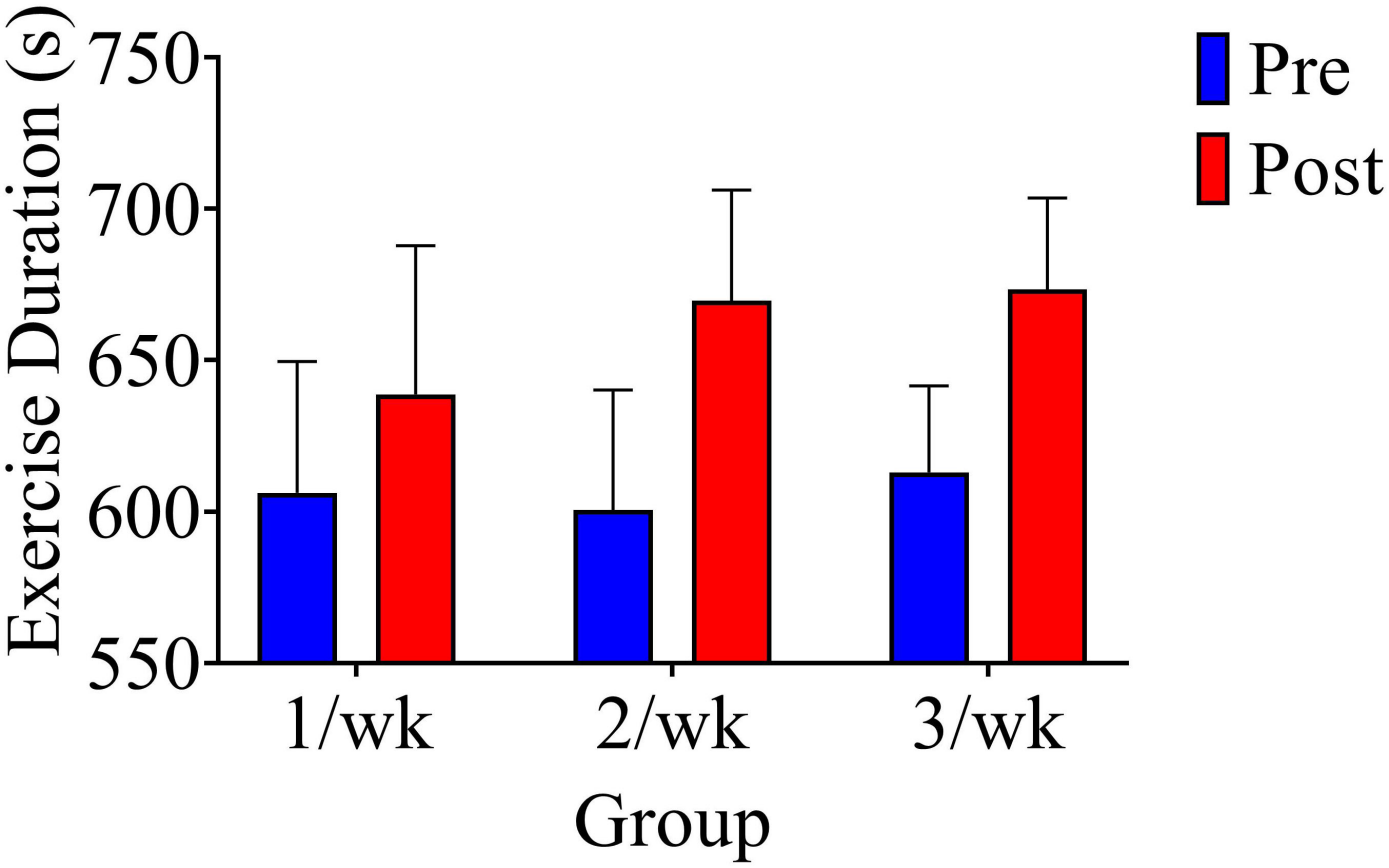
Changes in time to exhaustion before and after the intervention in each group. 1/wk, 2/wk, and 3/wk indicate 1, 2, and 3 sessions per week, respectively; Pre indicates pre−test and Post indicates post−test.

Time to exhaustion results are shown in **Figure 2**. The means ± SD before and after the intervention were as follows: in the 1−session group, 606.20 ± 43.36 s and 638.73 ± 49.08 s; in the 2−session group, 600.50 ± 39.63 s and 669.64 ± 36.55 s; and in the 3−session group, 612.91 ± 28.58 s and 673.42 ± 30.16 s, respectively. Repeated−measures ANOVA revealed a significant main effect of time (F(1, 38) = 45.954, p < 0.001, η² = 0.547), with no significant main effect of group (F(2, 38) = 1.663, p = 0.203, η² = 0.080) and no significant group × time interaction (F(2, 38) = 2.051, p = 0.143, η² = 0.097). *Post hoc* multiple comparisons with Bonferroni adjustment indicated that time to exhaustion was significantly longer at post-test than at pre-test (p < 0.001).

### Running economy test

#### Oxygen uptake

**Figure 3:**
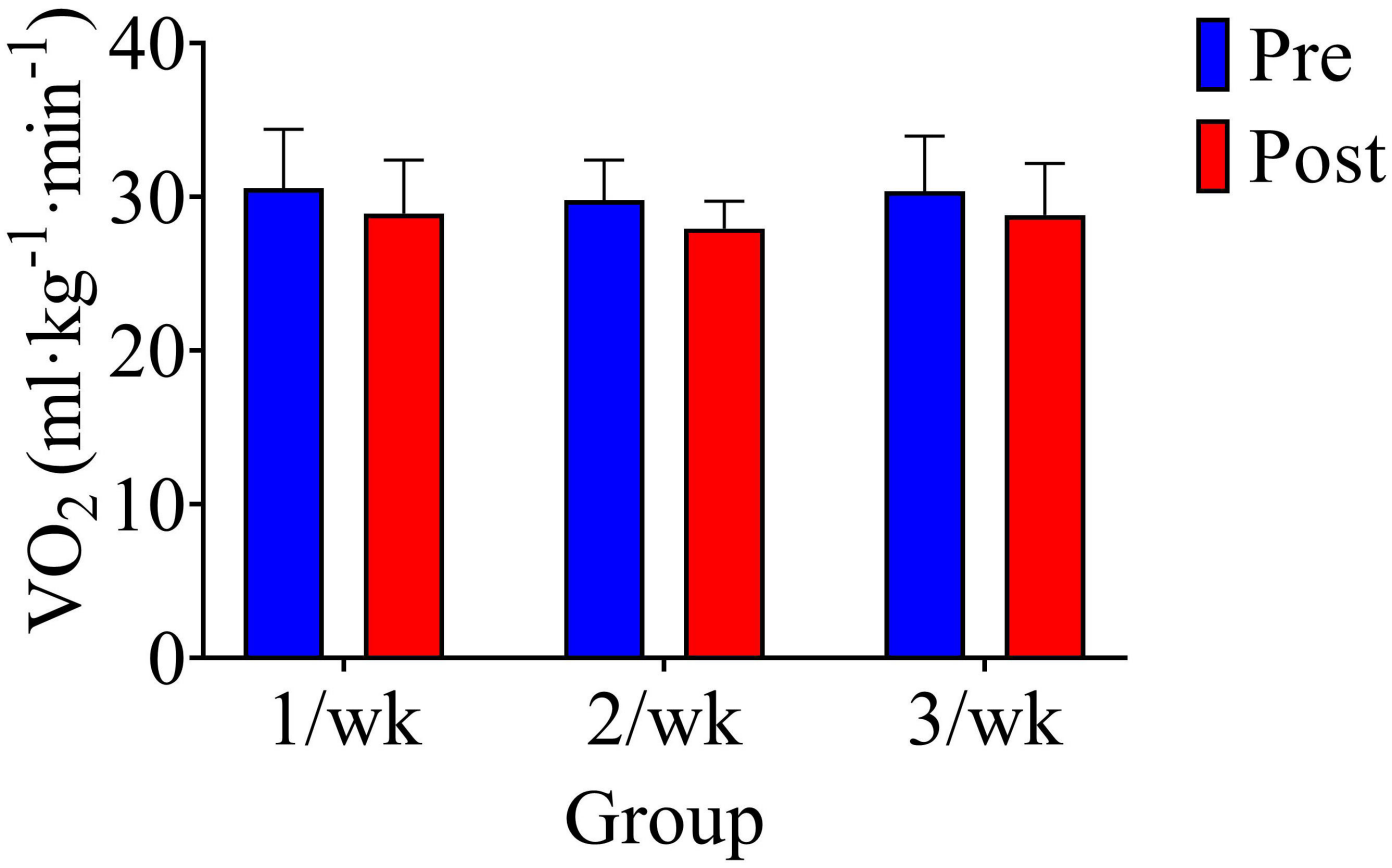
Changes in VO_2_ during the RE test before and after the intervention in each group. 1/wk, 2/wk, and 3/wk indicate 1, 2, and 3 sessions per week, respectively; Pre indicates pre−test and Post indicates post−test.

VO_2_ during the RE test is presented in **Figure 3**. The means ± SD before and after the intervention were as follows: in the 1−session group, 30.56 ± 3.82 ml·kg^-1^·min^-1^ and 28.92 ± 3.47 ml·kg^-1^·min^-1^; in the 2−session group, 29.78 ± 2.60 ml·kg^-1^·min^-1^ and 27.94 ± 1.77 ml·kg^-1^·min^-1^; and in the 3−session group, 30.35 ± 3.60 ml·kg^-1^·min^-1^ and 28.82 ± 3.36 ml·kg^-1^·min^-1^, respectively. Repeated−measures ANOVA showed no significant main effect of time (F(1, 38) = 4.043, p = 0.051, η² = 0.096), with no significant main effect of group (F(2, 38) = 1.001, p = 0.377, η² = 0.050) and no significant group × time interaction (F(2, 38) = 0.011, p = 0.989, η² = 0.001).

#### Heart rate

**Figure 4:**
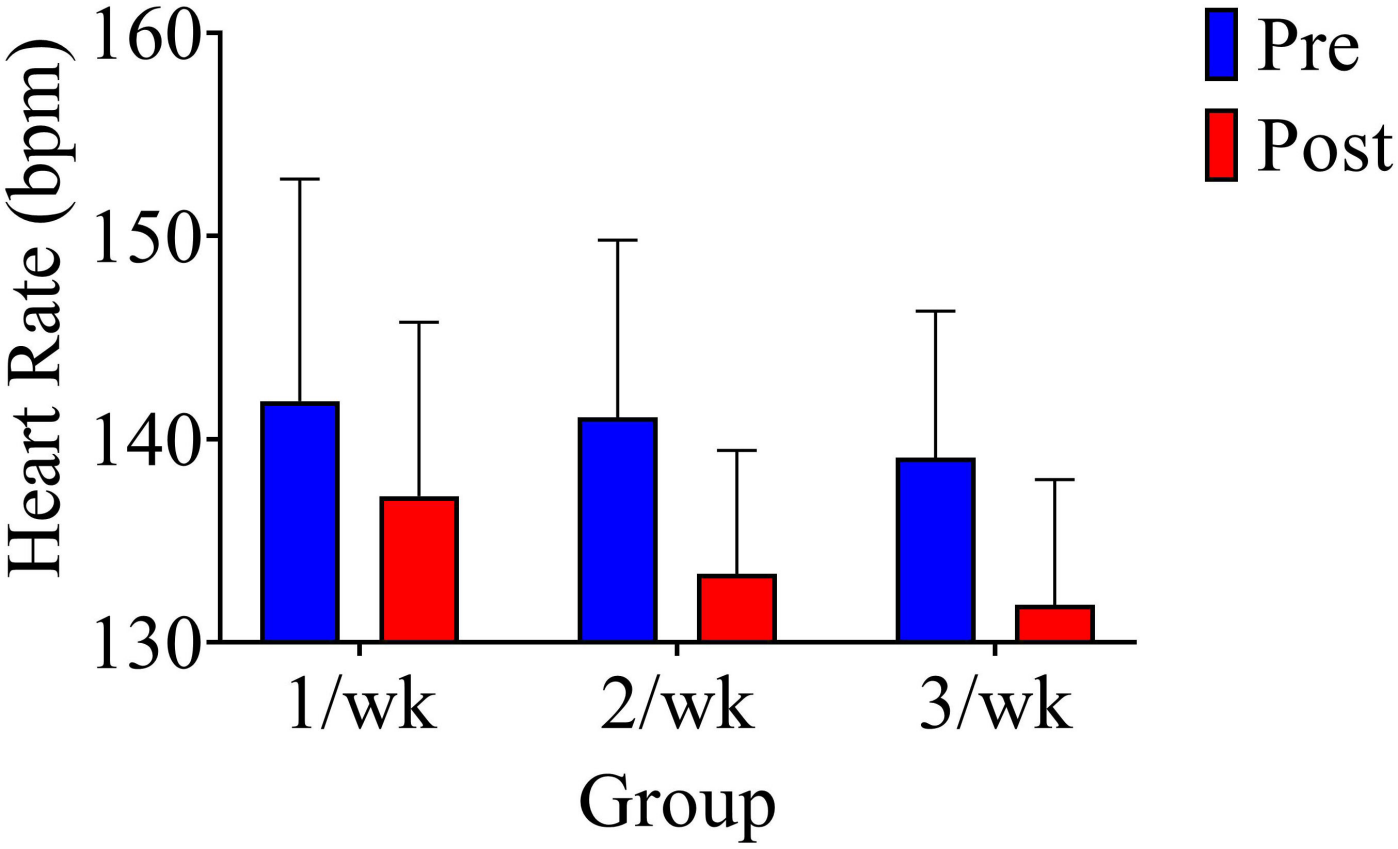
Changes in HR during the RE test before and after the intervention in each group. 1/wk, 2/wk, and 3/wk indicate 1, 2, and 3 sessions per week, respectively; Pre indicates pre−test and Post indicates post−test.

HR responses are shown in **Figure 4**. The means ± SD before and after the intervention were as follows: in the 1−session group, 141.87 ± 10.94 bpm and 137.20 ± 8.55 bpm; in the 2−session group, 141.07 ± 8.72 bpm and 133.35 ± 6.08 bpm; and in the 3−session group, 139.08 ± 7.22 bpm and 131.83 ± 6.18 bpm, respectively. Repeated−measures ANOVA indicated a significant main effect of time (F(1, 38) = 24.673, p < 0.001, η² = 0.394), with no significant main effect of group (F(2, 38) = 1.124, p = 0.336, η² = 0.056) and no significant group × time interaction (F(2, 38) = 0.551, p = 0.581, η² = 0.028). *Post hoc* multiple comparisons with Bonferroni adjustment indicated that HR was significantly lower at post-test than at pre-test (p < 0.001).

#### Ventilatory threshold

**Figure 5:**
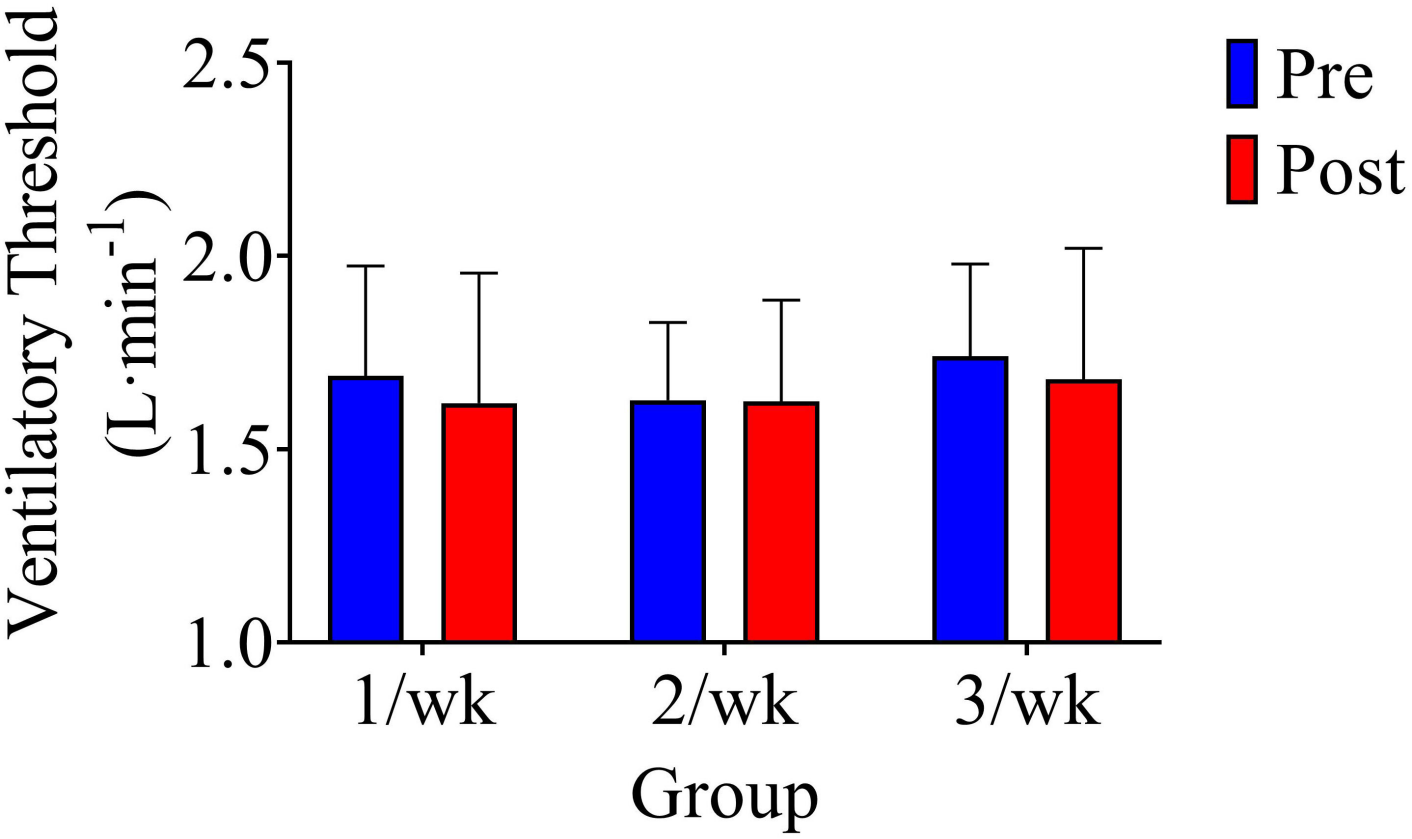
Changes in VT before and after the intervention in each group. 1/wk, 2/wk, and 3/wk indicate 1, 2, and 3 sessions per week, respectively; Pre indicates pre−test and Post indicates post−test.

VT results are presented in **Figure 5**. The means ± SD before and after the intervention were as follows: in the 1−session group, 1.69 ± 0.28 L·min^-1^ and 1.62 ± 0.34 L·min^-1^; in the 2−session group, 1.63 ± 0.20 L·min^-1^ and 1.62 ± 0.26 L·min^-1^; and in the 3−session group, 1.74 ± 0.24 L·min^-1^ and 1.68 ± 0.34 L·min^-1^, respectively. Repeated−measures ANOVA revealed no significant main effect of time (F(1, 38) = 0.489, p = 0.489, η² = 0.012), no significant main effect of group (F(2, 38) = 0.639, p = 0.534, η² = 0.033), and no significant group × time interaction (F(2, 38) = 0.114, p = 0.893, η² = 0.006).

#### Respiratory exchange ratio

**Figure 6:**
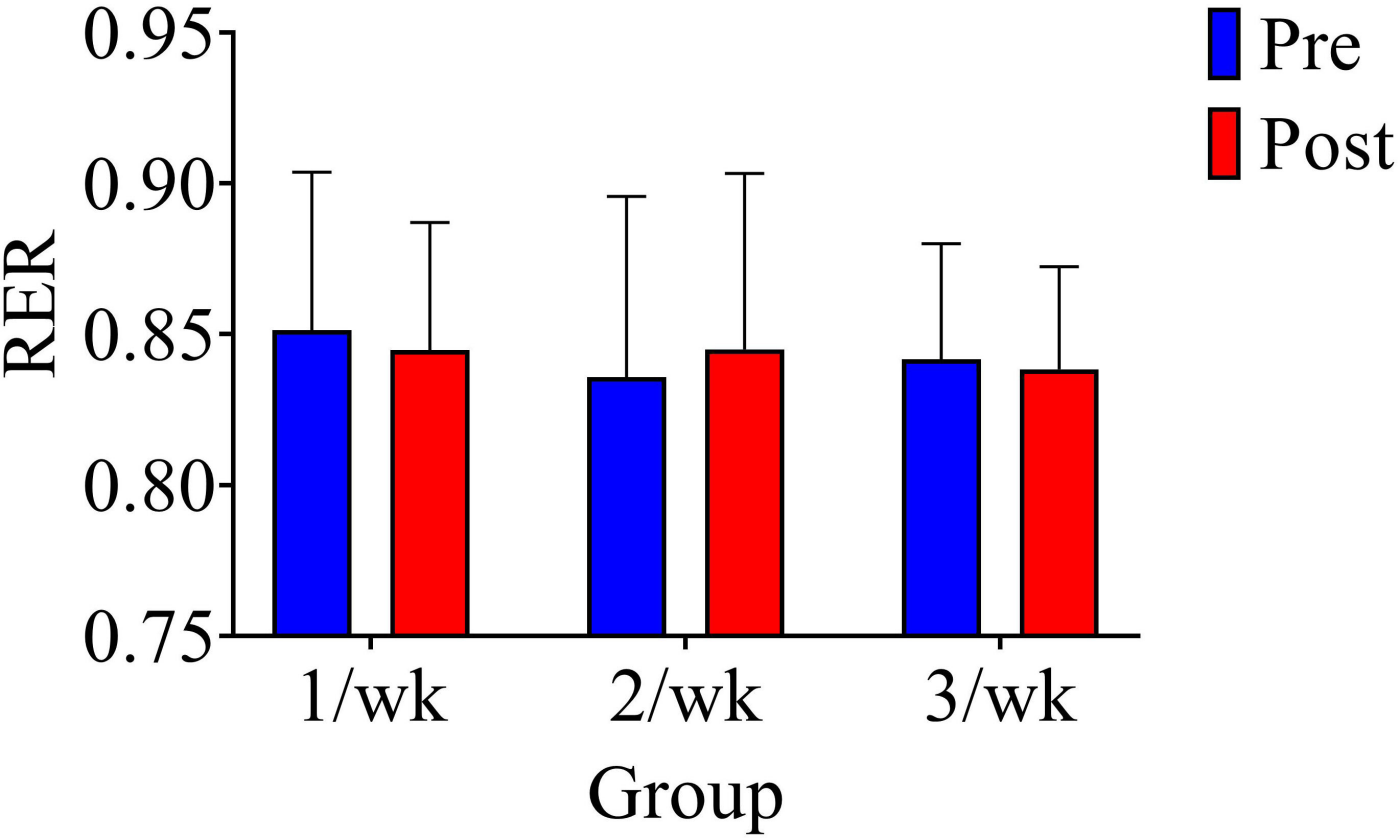
Changes in RER before and after the intervention in each group. 1/wk, 2/wk, and 3/wk indicate 1, 2, and 3 sessions per week, respectively; Pre indicates pre−test and Post indicates post−test.

RER results are shown in **Figure 6**. The means ± SD before and after the intervention were as follows: in the 1−session group, 0.85 ± 0.05 and 0.84 ± 0.04; in the 2−session group, 0.84 ± 0.06 and 0.85 ± 0.06; and in the 3−session group, 0.84 ± 0.04 and 0.84 ± 0.03, respectively. Repeated−measures ANOVA showed no significant main effect of time (F(1, 38) = 0.001, p = 0.984, η² = 0.001), no significant main effect of group (F(2, 38) = 0.279, p = 0.758, η² = 0.014), and no significant group × time interaction (F(2, 38) = 0.183, p = 0.834, η² = 0.010).

#### Blood lactate concentration

**Figure 7:**
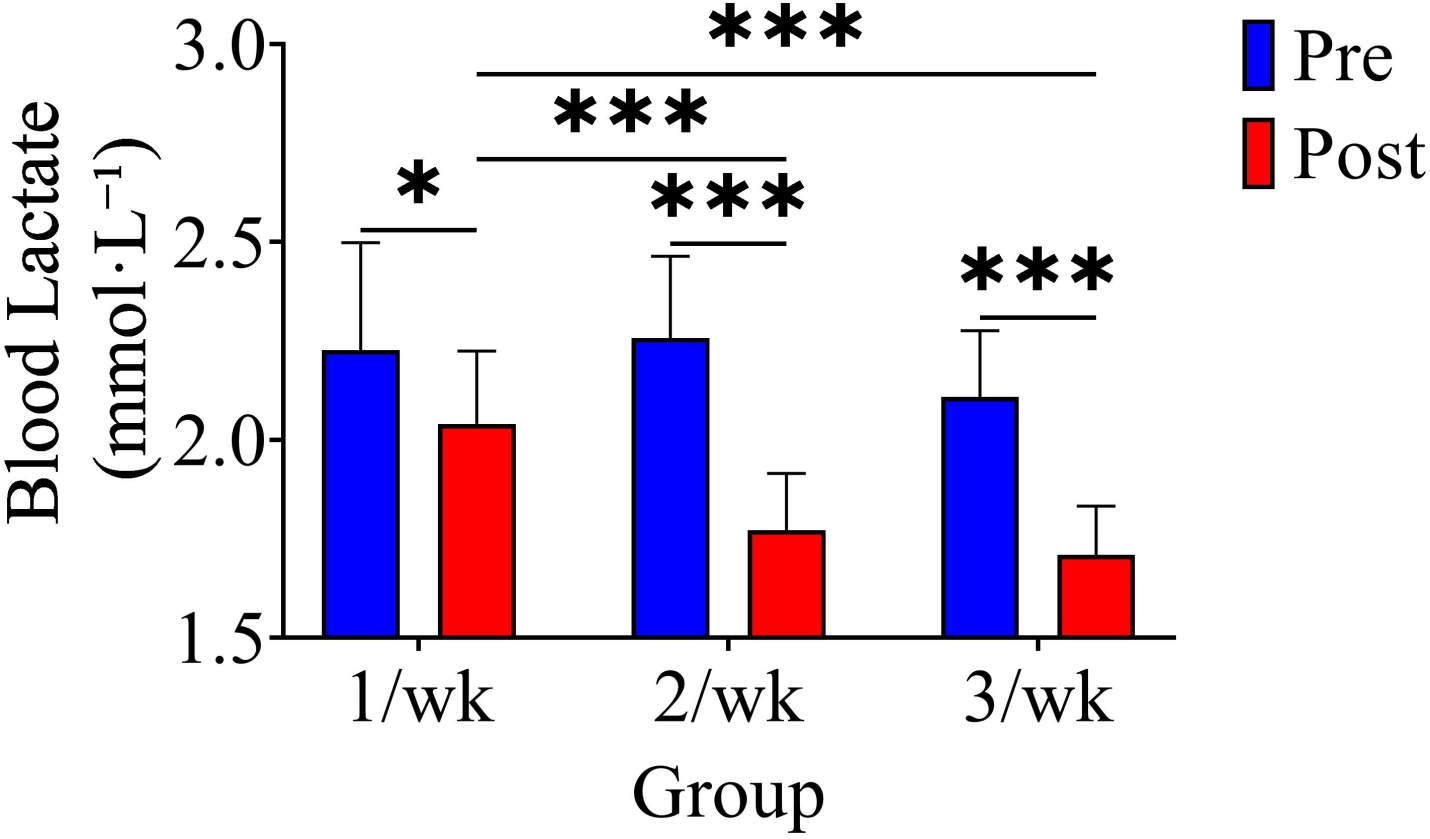
Comparison of blood lactate concentration before and after the intervention among the three groups. 1/wk, 2/wk and 3/wk represent 1 session/week, 2 sessions/week and 3 sessions/week, respectively; Pre indicates pre-intervention, Post indicates post-intervention; * indicates p < 0.05,*** indicates p < 0.001.

As shown in **Figure 7**, the mean blood lactate concentrations and standard deviations before and after the intervention were as follows: in the 1 session/week group, 2.23 ± 0.27 mmol·L^-1^ at pre-test and 2.04 ± 0.18 mmol·L^-1^ at post-test; in the 2 sessions/week group, 2.26 ± 0.21 mmol·L^-1^ at pre-test and 1.77 ± 0.14 mmol·L^-1^ at post-test; and in the 3 sessions/week group, 2.11 ± 0.17 mmol·L^-1^ at pre-test and 1.71 ± 0.12 mmol·L^-1^ at post-test. Repeated-measures ANOVA revealed a significant main effect of time (F(1, 38) = 71.649, p < 0.001, η² = 0.653), a significant main effect of group (F(2, 38) = 9.062, p < 0.001, η² = 0.323), and a significant group × time interaction (F(2, 38) = 4.745, p = 0.014, η² = 0.200). *Post hoc* pairwise comparisons were conducted with Bonferroni correction, setting the significance level at α = 0.017 (0.05/3). The results indicated that post-test values were significantly lower than pre-test values in all three groups (1/wk: p = 0.011; 2/wk: p < 0.001; 1/wk: p < 0.001);, and that post-test values in the 2 sessions/week and 3 sessions/week groups were significantly lower than those in the 1 session/week group (p < 0.001).

## Discussion

The present study examined male university students with regular exercise habits and compared the effects of 6 weeks of 4 × 4 min high−intensity interval training (HIIT) performed at three weekly frequencies (1, 2, and 3 sessions) on cardiorespiratory function, metabolic indices, and exercise performance. The training structure and intensity were consistent across groups, with only weekly frequency manipulated. The following sections analyze the findings based on the magnitude of changes and comprehensively evaluate the effectiveness and feasibility of the three training frequencies.

### Effects of different HIIT frequencies on VO_2_max

The present results showed an overall upward trend in VO_2_max across the three groups, with a significant main effect of time. In terms of improvement magnitude, the 2−session and 3−session groups exhibited similar substantial increases of approximately 5–6 ml·kg^-1^·min^-1^, corresponding to a ~10% relative improvement. In contrast, the 1−session group showed only a small upward shift, with a much smaller magnitude of change than the other two groups. This indicates that, under a 6-week 4 × 4 min HIIT program, moderate- and high-frequency training can induce marked improvements in aerobic capacity in male university students, whereas low-frequency training fails to elicit sufficient cumulative training effects.

The magnitude of VO_2_max improvements observed in the 2− and 3−session groups aligns with previous studies reporting significant aerobic adaptations following 4–8 weeks of HIIT in young healthy males and university students (Wen et al., 2019). This suggests that the 4 × 4 min HIIT protocol employed in this study is highly time-efficient for this population, yielding meaningful improvements in cardiorespiratory fitness within a short period.

Of note, the 3−session group exhibited a numerically lower baseline VO_2_max, which may confer a greater adaptive potential consistent with the principle of diminishing returns in trained individuals. While this subtle baseline difference might partly contribute to the larger relative improvement in the 3−session group, the similar absolute increases in VO_2_max between the 2− and 3−session groups suggest that training frequency remained the primary stimulus for adaptation. Furthermore, all participants were physically active young males, and randomization ensured that systematic between−group differences were minimized, reducing the risk of selection bias. It should be emphasized that, although VO_2_max increased significantly in both the 2 sessions/week and 3 sessions/week groups, neither the main effect of group nor the group × time interaction reached statistical significance. This suggests that, within a 6-week timeframe, increasing training frequency from 2 to 3 sessions per week did not result in greater improvements. This finding is generally consistent with the notion that a moderate HIIT frequency (approximately twice per week) may already achieve VO_2_max gains comparable to those of higher-frequency programs (Bacon et al., 2013). It may be inferred that once the total volume of high-intensity stimuli reaches a certain threshold, both central and peripheral adaptations of the cardiorespiratory system are largely established, and further increases in training frequency yield diminishing marginal returns.

By contrast, although the 1 session/week group followed the same 4 × 4 min HIIT format, the longer intervals between sessions likely prevented a stable cumulative adaptation from being established through repeated high-stress loads, resulting in only a modest increase in VO_2_max. From a training prescription perspective, for male university students with some training background who aim to achieve a marked increase in VO_2_max within approximately 6 weeks, a training frequency of at least 2 sessions per week appears necessary. Under the present conditions, further increasing frequency to 3 sessions per week did not provide clear additional benefits in VO_2_max.

### Changes in time to exhaustion and improvements in maximal exercise tolerance

Incremental treadmill testing revealed that time to exhaustion increased notably in all three groups post−intervention, with a significant main effect of time but no significant main effect of group or group × time interaction. These findings indicate that 6 weeks of HIIT can substantially improve maximal exercise tolerance under graded incremental load in male university students, regardless of whether the weekly frequency is 1, 2 or 3 sessions. This is reflected in a general enhancement of maximal exercise tolerance reserves.

On the one hand, increases in time to exhaustion are closely associated with improvements in VO_2_max (Garber et al., 2011). In the 2 sessions/week and 3 sessions/week groups, significant increases in VO_2_max were accompanied by marked extensions in time to exhaustion, suggesting that a higher maximal aerobic capacity enables participants to sustain higher workloads for longer durations and delay the point of volitional exhaustion. On the other hand, even the 1−session group, with relatively smaller improvements in VO_2_max, time to exhaustion still increased significantly. This implies that enhancements in maximal exercise tolerance are not entirely dependent on VO_2_max and may also be related to improved lactate tolerance; greater tolerance to dyspnea, muscle soreness and other discomforts; optimized perception of effort; and strengthened volitional and psychological attributes (Beneke et al., 2011; Noakes, 2012).

Through repeated exposure to intense stress characterized by high lactate and hyperventilation, HIIT can enhance both physiological and psychological tolerance to discomfort associated with maximal exercise, improve the perception and regulation of fatigue signals, and thereby enable participants to “hold on longer” during incremental tests (Iaia and Bangsbo, 2010). Simultaneously, HIIT exerts a reinforcing effect on the neuromuscular system, improving movement coordination and force output efficiency during high-intensity exercise, which may indirectly reduce the biomechanical load at a given running speed (Berryman et al., 2018).

It is noteworthy that, although there were some differences in the magnitude of improvement in time to exhaustion among the three groups, these did not translate into statistically significant differences between training frequencies. This suggests that, within the present training load and duration, the presence or absence of continued HIIT may have a greater impact on maximal exercise tolerance than subtle differences in frequency within the range of 1–3 sessions per week. From a practical standpoint, even one structured HIIT session per week can meaningfully increase time to exhaustion during maximal exercise.

### Changes in oxygen uptake, ventilatory threshold and respiratory exchange ratio at submaximal intensity and their implications

Under the 9 km/h steady-state running condition, this study used VO_2_, VT and RER to examine changes in RE at submaximal intensity. Overall, these metabolic indices showed relatively modest responses to the 6-week HIIT intervention and did not exhibit changes of similar magnitude to those observed in VO_2_max and blood lactate. This suggests that short-term high-intensity interval loading has limited impact on “structural RE,” although the trends remain informative.

Regarding VO_2_, oxygen consumption at 9 km/h showed a mild overall downward tendency across groups. However, neither main effect nor interaction effect revealed significant differences. Thus, at the group level, there was a general trend toward slightly reduced oxygen cost per unit speed, but large inter-individual variability and small absolute changes likely prevented stable statistical differences from emerging within or between groups. For physically active young men such as those in this study, baseline VO_2_max and RE are already relatively high, and 9 km/h represents a relatively low relative intensity. Oxygen consumption at this fixed speed is already economical, leaving limited “room” for further substantial reductions and predisposing to a “ceiling effect” (Midgley et al., 2007). In addition, the 4 × 4 min HIIT protocol is primarily designed to enhance maximal cardiorespiratory and metabolic capacity, with limited direct emphasis on technical aspects such as stride length and frequency, and the coordination of stance and swing phases, which are often critical for large reductions in oxygen cost at a given speed (Saunders et al., 2004). This partly explains the phenomenon whereby subjects may feel subjectively less effortful while VO_2_ at fixed speed changes little.

Similarly, changes in VT did not reach statistical significance, which to some extent corroborates the VO_2_ findings. VT reflects the transition point at which the body shifts from predominantly aerobic energy supply to a greater contribution from anaerobic glycolysis and is often regarded as an important physiological basis for the intensity that can be sustained for prolonged periods in endurance exercise (Beaver et al., 1986). In this study, although the 2− and 3−session groups exhibited larger VO_2_max gains and reduced blood lactate at 9 km/h, VT improvements were modest. One explanation is that participants were already regularly physically active before enrolment, so the relative proportion of VT to VO_2_max may have been relatively high at baseline, making further short-term gains difficult. Another possibility is that the 4 × 4 min HIIT protocol concentrated loading in intensity domains clearly above VT, thereby favoring improvements in maximal cardiorespiratory capacity and high-intensity tolerance, while providing relatively insufficient targeted stimuli for “prolonged work capacity near VT” (Jones and Carter, 2000). As a result, VT may not have been sufficiently driven to shift upwards. In addition, VT determination is sensitive to testing protocols, evaluation methods and assessor experience; when true physiological changes are small and sample size is limited, statistical differences are harder to detect.

RER remained stable at approximately 0.84–0.85 before and after the intervention, with no significant main effects of time, group or interaction, indicating that the relative contributions of fat and carbohydrate to energy supply at 9 km/h were broadly similar within and between groups. Combined with the significant decline in blood lactate, this suggests that, after HIIT, reliance on anaerobic glycolysis at the same speed was reduced and the capacity of the aerobic system to clear and reutilize lactate was enhanced. However, against a background of predominantly aerobic intensity with relatively balanced carbohydrate–fat oxidation, this subtle reduction in the anaerobic contribution was not sufficient to manifest as a marked change in RER at the macro level. In other words, under the moderate-to-low intensity conditions selected in this study, RER does not appear to be a highly sensitive index for detecting short-term HIIT-induced changes in RE. Its lack of significant change does not negate potential improvements in metabolic economy, but rather indicates that fine-tuning of substrate utilization is more readily captured by indices such as blood lactate that more directly reflect the degree of anaerobic glycolytic involvement.

Taking into account the characteristics of the participants, the training duration and the testing speed, the influence of short-term HIIT on “structural RE indices” such as VO_2_, VT and RER at 9 km/h appears limited, with no large, uniform group-level decreases or upward shifts observed. This suggests that, over 6 weeks, the 4 × 4 min HIIT protocol primarily improves the subjective experience of “how easy it feels to run” by enhancing maximal aerobic capacity and reducing circulatory and lactate metabolic burden, rather than by substantially lowering fixed-speed oxygen cost or clearly shifting VT. To achieve more pronounced improvements in fixed-speed VO_2_ and VT, a longer training period and/or a combination of HIIT with moderate-intensity continuous running and targeted running technique training may be required.

### Heart rate and blood lactate responses as reflections of circulatory and metabolic economy

Compared with the above “structural economy indices,” the present findings indicate that HR and blood lactate concentration at 9 km/h were more sensitive to the 6-week HIIT intervention and more clearly reflected improvements in circulatory and metabolic economy.

With respect to HR, exercise HR at 9 km/h decreased markedly post−intervention across groups, with a clear main effect of time, but no significant main effect of group or group × time interaction. The 2−session group showed the largest reduction, followed by the 3−session group, with the 1−session group demonstrating the smallest decrease. These findings confirm that HIIT reduced submaximal HR at 9 km/h, with twice− and thrice−weekly training yielding more substantial improvements and greater cardiovascular efficiency. The underlying physiological mechanisms likely include enhanced myocardial contractile function, increased stroke volume, and improved ventricular compliance and filling efficiency induced by repeated high-intensity loading, allowing the necessary cardiac output for 9 km/h running to be maintained at a lower HR (Levy et al., 1998; Wisløff et al., 2007). In addition, autonomic regulation may be optimized, with increased parasympathetic activity and attenuated sympathetic drive during submaximal exercise, resulting in a downward shift in HR responses (Sandercock et al., 2005). For male university students, this implies reduced sensations of breathlessness and palpitations at a given running speed, improved exercise comfort and potentially greater psychological acceptance of, and willingness to engage in, sustained aerobic exercise.

Blood lactate concentration provided a more intuitive metabolic reflection of HIIT-induced improvements in RE. In this study, blood lactate levels measured after 9 km/h steady-state running decreased significantly over time in all three groups, with a significant main effect of time, as well as significant main effects of group and group × time interaction. Post-intervention blood lactate levels in the 2 sessions/week and 3 sessions/week groups were significantly lower than in the 1 session/week group. These findings indicate that HIIT can substantially reduce lactate accumulation and the contribution of anaerobic glycolysis at the same submaximal workload, with moderate- and high-frequency training producing greater improvements than low-frequency training.

Mechanistically, HIIT promotes mitochondrial biogenesis and increases oxidative enzyme activity in skeletal muscle, thereby enhancing aerobic energy production capacity. At the same time, it can upregulate the expression of lactate transporters (e.g., MCT-1, MCT-4), improving the transport and reutilization of lactate from working muscles to surrounding tissues and organs such as the heart and liver, so that at the same submaximal workload lactate production is reduced and clearance accelerated (Bonen, 2001; Gibala et al., 2006). The significant decrease in blood lactate observed in this study indicates that, during 9 km/h running, the body relies more heavily on aerobic metabolism to meet energy demands, with a reduced relative contribution from anaerobic pathways, thereby lowering metabolic stress and post-exercise recovery demands. From a metabolic standpoint, this directly reflects improved RE.

It should be noted that, although the 3 sessions/week group showed slightly greater improvements in blood lactate than the 2 sessions/week group, VO_2_max and other key indices did not differ substantially between the two. This suggests that further increasing frequency beyond twice weekly may yield only limited marginal gains in central aerobic capacity, while still offering some additional peripheral metabolic adaptations. However, these extra metabolic benefits are obtained at the cost of greater training stress and a marked reduction in completion rate. This underscores the need to balance metabolic adaptation benefits against adherence, fatigue and recovery when prescribing training frequencies in practice.

Taken together, changes in HR and blood lactate indicate that short-term HIIT improves RE at submaximal intensity primarily by “economizing” circulatory and metabolic load—that is, reducing the cardiovascular and lactate metabolic stress required to sustain a given speed—rather than solely by lowering fixed-speed oxygen cost or substantially shifting VT. Such changes, whereby running feels “less taxing and more economical,” have more direct practical relevance for the daily running experience and long-term exercise adherence of male university students.

### Overall evaluation of training frequency, completion and comprehensive outcomes

In parallel with physiological indices, this study also recorded the number of participants who completed the intervention in each group. The results showed that all participants in the 1 session/week group completed the intervention (completion rate 100%), whereas one participant withdrew from the 2 sessions/week group (completion rate ≈ 93%) and the 3 sessions/week group had the highest number of withdrawals, with a completion rate of approximately 80%. Completion rates declined as training frequency increased, suggesting that higher-frequency HIIT was perceived as more burdensome and more detrimental to adherence in this population of male university students.

Integrating physiological benefits with completion data allows a clearer evaluation of the three frequency schemes:

Once−weekly HIIT: Low load, high adherence, and improvements in time to exhaustion, submaximal HR, and lactate. However, improvements in key indices (VO_2_max, HR, time to exhaustion) were modest, limiting gains in core aerobic capacity.

Thrice−weekly HIIT: Comparable or marginally greater improvements in VO_2_max, time to exhaustion, HR, and lactate versus twice−weekly training, but minimal additional benefits. Higher training stress and lower completion rates reduced feasibility for male university students with academic and recovery constraints.

Twice−weekly HIIT: Relatively optimal balance. Substantial improvements in VO_2_max, time to exhaustion, submaximal HR, and lactate, alongside high completion rates (only marginally lower than once−weekly). Post−intervention lactate was significantly lower than in the once−weekly group, confirming favorable balance between efficacy and tolerability.

Considering both physiological responses and completion rates, the present findings suggest that, within this population and over a 6-week training period, increasing frequency from 2 to 3 sessions per week does not yield clearly superior benefits in key physiological indices but substantially increases training stress and the risk of dropout. In contrast, while 1 session/week is associated with the best adherence, it provides insufficient stimulus for meaningful improvements in VO_2_max. Therefore, twice-weekly 4 × 4 min HIIT appears to be the optimal training frequency among the three schemes, as it enhances VO_2_max, time to exhaustion and RE–related indices such as submaximal HR and blood lactate, while maintaining a relatively high completion rate and good feasibility. This conclusion provides direct empirical evidence for prescribing HIIT to improve cardiorespiratory endurance and RE in male university students, suggesting that a twice-weekly frequency should be prioritized in practice to balance training benefits with load tolerability and long-term adherence.

### Limitations

Several limitations of this study should be acknowledged when interpreting the results. First, the participants were physically active male university students from a single institution, with a relatively small sample size. Women and sedentary individuals were not included, which limits the generalizability of the findings. Responses to different HIIT frequencies may vary by sex, age and baseline fitness level. Second, the 6-week intervention period represents a short-to-medium training duration, which is well suited to detecting changes in “fast-responding” variables such as VO_2_max, time to exhaustion and blood lactate, but less so for “slow-adapting” factors related to RE, such as muscle–tendon compliance and gait mechanics. As a result, the long-term effects of different HIIT frequencies on RE may have been underestimated. Third, the study primarily evaluated training effects using cardiorespiratory and metabolic indices and did not incorporate cardiac structural and functional imaging, muscle biochemical markers or molecular-level measurements. This makes it difficult to elucidate the specific central and peripheral mechanisms underlying frequency-dependent differences in HIIT responses. Finally, although the number of completers and completion rates were recorded, subjective variables closely linked to adherence—such as perceived fatigue, muscle soreness, sleep quality and psychological stress—were not systematically quantified. Consequently, the overall balance among training load, recovery and daily life was not fully captured, which to some extent restricts the comprehensiveness of the assessment of optimal training frequency.

## Conclusion

Six weeks of 4 × 4 min high−intensity interval training markedly prolonged time to exhaustion and reduced submaximal heart rate and blood lactate concentration in male university students. In terms of improvement magnitude, both twice−weekly and thrice−weekly training elicited substantial increases in maximal oxygen uptake (VO_2_max) and pronounced reductions in submaximal blood lactate, while once−weekly training yielded relatively modest improvements in these indices. Within the scope of this study, twice−weekly and thrice−weekly training resulted in comparable improvements in most cardiorespiratory and metabolic variables, although the completion rate was notably lower in the thrice−weekly group. Considering intervention efficacy, magnitude of physiological improvements, and training adherence, twice−weekly 4 × 4 min HIIT demonstrated a relatively favorable balance between training benefits and participant tolerability under the specific conditions of this study. Given the limitations of a relatively small sample size, short intervention duration, and a homogeneous cohort of physically active male university students, the generalizability of these findings is constrained. Further validation in larger, diverse populations and over longer periods is warranted.

## Data Availability

The original contributions presented in the study are included in the article/**Supplementary Material**. Further inquiries can be directed to the corresponding author.
